# Exposure to a Titanium Dioxide Product Alters MicroRNA Expression in Human Cells

**DOI:** 10.3390/toxics14040276

**Published:** 2026-03-25

**Authors:** Shivangi Shrimali, Carlos Wells, Marta Pogribna, Beverly Word, Paul Rogers, Beverly Lyn-Cook, George Hammons

**Affiliations:** 1 Division of Bioinformatics and Biostatistics, FDA/National Center for Toxicological Research, Jefferson, AR 72079, USA; 2 Division of Biochemical Toxicology, Jefferson, AR 72079, USA

**Keywords:** miRNA expression, titanium dioxide, human cells, nanotoxicity

## Abstract

The safety of titanium dioxide (TiO_2_), widely used in foods and personal care products, has been of on-going concern. Adverse effects of TiO_2_ have been reported, suggesting risk to human health. To evaluate its potential epigenotoxicity, the effect of exposure to a TiO_2_ product, to which humans could be exposed, on microRNA (miRNA) expression (a primary epigenetic mechanism) was investigated using human cell lines (Caco-2, HCT116 (colorectal) and HepG2, SNU387 (liver)) relevant to human exposure. The effect of TiO_2_ nanomaterial exposure on expression levels of miRNA was determined using the TaqMan Array Human microRNA A+B Card Set v3.0 platform. Differentially expressed miRNAs were identified (SNU387 (n = 112), HepG2 (n = 97), Caco-2 (n = 94), and HCT116 (n = 53)). Kyoto Encyclopedia of Genes and Genomes (KEGG) and Gene Ontology (GO) functional enrichment analysis of target genes provided insights into the roles of modulating pathways, which can be associated with diseases. Top 10 KEGG pathways in each cell line included MAPK signaling pathway, Axon guidance, cell cycle, Hippo signaling pathway, and Endocytosis. Findings from the study clearly demonstrate the impact of TiO_2_ exposure on miRNA expression, supporting the potential involvement of this epigenetic mechanism in its biological responses. Hence, epigenetic studies are important for the complete assessment of the potential risk from exposure.

## 1. Introduction

Nanosized titanium dioxide (TiO_2_) is one of the three most produced nanomaterials and is second only to silver nanoparticles in terms of the most advertised nanomaterial in consumer products [1]. Given its brightening/whitening properties, there is widespread use of TiO_2_ in the food industry, as well as in personal care products, including shampoos, lip balms, deodorants, toothpastes, and sunscreens [2]. The material is referred to as INS171 in North America and as E171 in Europe. In the USA, TiO_2_ can be used in food if its content is less than 1% of the total weight of the product without the requirement of ingredient label disclosure [3]. Chewing gum, a variety of sweets, baked goods, confectionery products, ice cream, and beverages are among the various food stuffs using TiO_2_ [4,5,6,7,8,9]. Candies, chewing gum, and sweets contain the highest TiO_2_ content, reaching 2.5 mg titanium (Ti)/g of food.

Human exposure to TiO_2_ has been estimated in several populations in the USA, UK, Netherlands, and China [4,10,11]. Results from these studies indicate that exposure levels differ among regions and are higher in young children compared to other age groups. In a recent study, a systematic search and meta-analysis was conducted to assess oral TiO_2_ exposure across different age groups and regions [12]. The mean oral TiO_2_ intake was found to range from 0.045 to 10.5 mg/kg body weight (bw)/day. A lifelong weighted average exposure was 1.43 mg/kg_bw_/day. Standardized Mean Differences between children and adults were 1.05 (general comparison) and 2.15 (ages 3–9 vs. 18–64). The long-term, repeated exposure to such quantities of TiO_2_ may result in tissue accumulation [13].

TiO_2_ in various products has been noted as a possible public health concern [2,14]. TiO_2_ can consist of nanoparticles and microparticles as a consequence of its production process, which unintentionally generates particles of a broad size distribution [4,15,16,17]. Although particular concerns regarding its potential impact on human health have been raised due to the presence of nanoparticles in the material, the importance of assessing the TiO_2_ product as a whole has been noted, given the substantial difference between the reference material P25 and products to which humans are exposed in their diet and use of various personal products [18,19,20]. Several reports with TiO_2_ products found no adverse effects. In contrast, other studies investigating the effects in vivo and in vitro in multiple animal and cell models have reported potential adverse effects, including inflammation, induction of reactive oxygen species (ROS), genotoxic effects, and hepatotoxic effects, among others [21,22,23,24].

Most studies on the toxicity mechanisms of nanomaterials have focused on the effects of these materials at the proteome and transcriptome levels in cells. There is, however, a need to also understand the epigenetic action of potential toxicants. Epigenetics is defined as “a stably heritable phenotype resulting from changes in a chromosome without alterations in the DNA sequence” [25]. One of the major epigenetic mechanisms is microRNA (miRNA) expression. miRNAs are endogenous, short (~22 nucleotides), non-coding RNAs regulating gene expression at post-transcriptional and translational levels via complementary base-pairing with target mRNA [26,27]. miRNAs play an essential role in a broad range of cellular processes, including differentiation, proliferation, apoptosis, and stress response [26]. High complementarity of miRNA and its mRNA target results in mRNA cleavage through an RNA interference mechanism. In contrast, partial complementarity, which is commonly seen in mammalian cells, results in translational inhibition. Single miRNAs can simultaneously regulate multiple targets or even whole biological networks [27]. The aberrant expression of miRNAs has been linked to various human diseases, including cancer, heart disease, and Alzheimer’s disease [28,29,30,31]. Increasing evidence shows that nanomaterials can induce changes in miRNA expression [32,33,34], thereby understanding the epigenotoxicity of these nanomaterials associated with miRNA expression is important in assessing their potential for harm.

In earlier studies, exposure to TiO_2_ nanoparticles models, including P25, has been reported to induce alterations in miRNA expression in target tissues and cells, thus indicating that the epigenetic changes induced by TiO_2_ nanoparticles may play a role in the potential harm of these particles to human health [35,36,37,38,39,40,41,42,43,44,45]. However, reports including a commercially available TiO_2_ product to which humans may be exposed in their diet have been limited. In the only study found, microarray analysis demonstrated that expression of miRNAs was affected following exposure to E171 in human stomach epithelial AGS cells [46]. The present study investigated the effects of such a nanomaterial in four human cell lines. The assessment included microarray analysis of differentially expressed miRNAs and bioinformatic analysis to provide insights into the biological processes and precise pathways that may be involved. The results from the current study found that exposure to the nanomaterial altered miRNA expression and that several biological processes and pathways can be affected, supporting the involvement of miRNA expression in the potential toxicity of this material and the critical need for its epigenetic assessment.

## 2. Materials and Methods

### 2.1. Nanomaterial Source and Characterization

TiO_2_ was supplied by Pure Organic Ingredients (Linton, UT, USA) (product described as exceptionally pure) and purchased from Amazon.com (Seattle, WA, USA). Characterization of the nanomaterial employed scanning electron microscopy–energy dispersive X-ray spectroscopy (SEM-EDS), X-ray diffraction (XRD), laser diffraction, Raman spectroscopy, and transmission electron microscopy (TEM), as previously reported [47]. Briefly, the TiO_2_ sample was analyzed for morphology, crystalline structure, particle size distribution, elemental composition, hydrodynamic diameter, and zeta potential. The SEM image of the powder TiO_2_ sample showed that it comprises generally spherical nanoparticles of heterogeneous size distribution. XRD results demonstrated that the crystalline form of the TiO_2_ sample is anatase, which was confirmed by the Raman spectral signals. Based on SEM-EDS, the TiO_2_ sample showed the predominant presence of Ti and O. Small amounts of Au, Pd, and C were also seen due to sample preparation methodology. No other elements were detected. Heterogeneous size distribution was observed with TEM. The size distribution histogram showed an average size of 242.3 ± 93.6 nm; nanoparticles and microparticles are present. The hydrodynamic size distribution of the TiO_2_ sample, using laser diffraction, indicated significant agglomeration in water and severe agglomeration in media. The zeta potential measurement of the sample, measured in water (pH 6.4) and 10 mM aqueous sodium chloride (pH 6.8) solutions, showed that the sample has an average zeta potential value of −42.4 and −41.6 mV, respectively. Zeta potential in 2.5 mM and 5 mM NaCl solution was −44.1 and −46.8 mV, respectively. Using Caco-2 cells as a model cell line, TEM revealed the uptake of the TiO_2_ particles in the cells after 24 h. The presence of the particles was further verified and confirmed by EDS.

### 2.2. Cell Lines and Treatment Conditions

Human cell lines, Caco-2, HCT116, HepG2, and SNU387, were obtained from the American Type Culture Collection (Manassas, VA, USA). The cells were cultured in growth media as recommended by the supplier and routinely maintained at 37 °C in a humidified 5% CO_2_ atmosphere. Cells were seeded in plates (5 × 10^5^ cells per plate) and subsequently treated with 10 µg/mL or 100 µg/mL TiO_2_ for 24 or 72 h, as in a previous study [47]. Cells were harvested and processed for further analysis at appropriate times.

### 2.3. miRNA Expression Array Analysis

The TaqMan Array Human microRNA A+B Card Set v3.0 (Applied Biosystems; Carlsbad, CA, USA) platform was used to compare miRNA expression levels between treated and control cells. The set provided an accurate quantitative value of 754 human microRNAs. Each card also contains three endogenous controls (Mamm-U6, RNU-44, and RNU-48) and a negative control (ath-miR-159a). Total RNA was isolated from treated and control cells using miRNeasy Mini kits (Qiagen; Valencia, CA, USA). Single-stranded cDNA was synthesized from total RNA samples using a TaqMan MicroRNA Reverse Transcription (RT) Kit (Applied Biosystems) and Megaplex^TM^ RT primers (Human Pool Set v3.0; Applied Biosystems) following the preamplification path per manufacturer’s protocols. cDNA samples were loaded on cards and run on a QuantStudio^TM^ 7 Flex Real Time PCR System (Applied Biosystems) according to the manufacturer’s protocols. Data are reported as fold change between the treated sample and control. If the fold change was <1, −1/(value) was calculated. To enable the comparison of expression patterns of miRNAs across experimental conditions, miRNA expression data were row-scaled (z-score normalization) in the heat maps. Hierarchical clustering was performed using Euclidean distance in R studio 4.3.1 (pheatmap package).

### 2.4. Bioinformatic Analysis of Differentially Expressed miRNAs

To investigate the functional involvement of miRNA after TiO_2_ treatment, target identification in R studio 4.3.1 was performed. For each differentially expressed miRNA across the four cell lines, validated targets were obtained using the *mutliMiR R package* (v. 1.24.0), which contains experimentally validated miRNA-target interaction databases such as miRTarBase, TarBase, and DIANA-TarBase. The output of the target identification step was used for pathway and gene ontology analyses.

Pathway enrichment was performed using the *clusterProfiler R package* (v. 4.10.1) (*enrichKEGG()* for KEGG (Kyoto Encyclopedia of Genes and Genomes) pathway analysis and *enrichGO()* for GO (gene ontology) analysis). KEGG human pathway database was used for over-representation analysis. Enrichment *p*-values were calculated using hypergeometric testing. Pathways were considered significantly enriched if *p* < 0.05 and ranked based on adjusted *p*-values. Gene ontology (GO) analysis was conducted using the enrichGO() function from the *clusterProfiler R package*. Target gene symbols were converted to Entrez IDs using the *org.Hs.eg.db database* (v. 3.18.0) GO enrichment was conducted separately for the Biological Process (BP), Cellular Component (CC), and Molecular Function (MF) ontologies, applying the Benjamini–Hochberg correction to control for false discovery. The top 5 enriched GO terms were visualized to identify biological functions potentially regulated by TiO_2_-responsive miRNAs.

## 3. Results

### 3.1. Differentially Expressed miRNAs

The effect of the TiO_2_ nanomaterial exposure on the expression levels of miRNA in the colon and liver cell lines was determined using the TaqMan Array Human microRNA A+B Card Set v3.0 platform. As shown in Table 1, there was a variation in the number of differentially expressed miRNAs across cell lines, with SNU387 having the highest number (n = 112), followed by HepG2 (n = 97), Caco-2 (n = 92), and HCT116 (n = 53).

**Figure 1:**
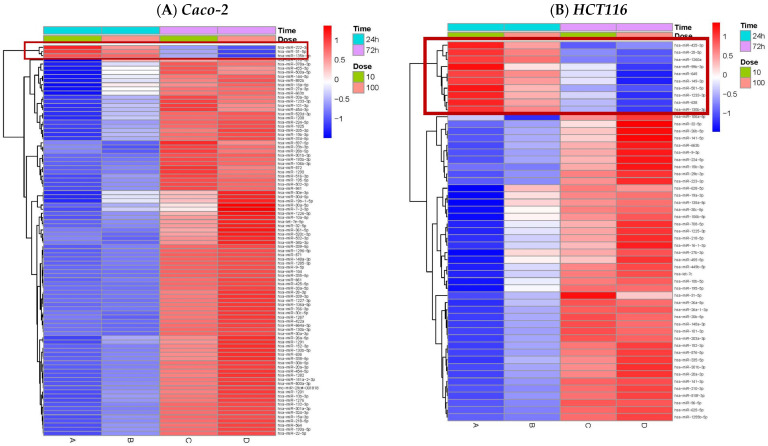

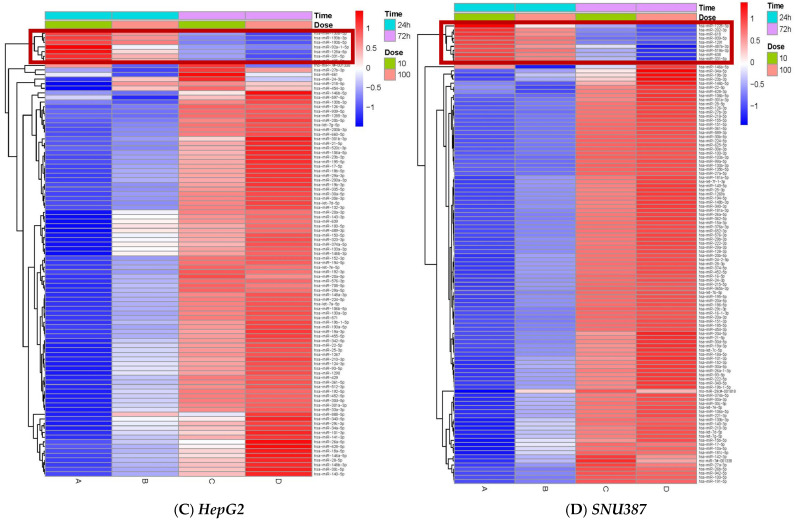
**Heatmaps showing hierarchical clustering of differentially expressed miRNAs following TiO_2_ treatment (10 µg/mL or 100 µg/mL TiO_2_ for 24 or 72 h) in four human cell lines.** Expression levels of miRNAs were determined using the TaqMan Array Human microRNA A+B Card Set v3.0 (Applied Biosystems; Carlsbad, CA, USA) platform. Each panel displays z-score normalized expression values, with red indicating upregulation and blue indicating downregulation relative to untreated controls. (**A**): Caco-2; (**B**): HCT116; (**C**): HepG2; (**D**): SNU387. Columns represent different treatment conditions (dose and time), and rows represent individual miRNAs. Clustering highlights distinct patterns of miRNA regulation across dose and time points for each cell line.

To compare the expression of each miRNA across different doses and time (10 or 100 µg/mL TiO_2_ for 24 or 72 h), a heatmap was generated using z-score normalization for each cell line. Row clustering was then performed to identify the miRNAs that exhibit distinct expression patterns across treatment conditions. As shown in Figure 1, Caco2 cells have two major clusters, one showing upregulation at the earlier exposure time (24 h) and one showing upregulation at the later exposure (72 h), regardless of TiO_2_ dose. Similar patterns were observed in HepG2, HCT116, and SNU387 cells, where clustering was dependent on both dose and time. Interestingly, a few miRNAs followed expression patterns independent of dose or exposure time; for example, hsa-miR-888-5p in HepG2, hsa-miR-146a-5p in SNU387, hsa-miR-27b-5p in Caco2, and hsa-miR-628-5p in HCT cells, among others were evident in the heatmaps. Several miRNAs show a similar pattern across different cell lines. Notably, miR-425-3p shows a comparable pattern in HCT116 and HepG2 cell lines, miR-638 displayed similar pattern in HCT116 and SNU387 cells, and miR-331-5p demonstrated consistent expression between HepG2 and SNU387 cells. These unique expression trends warrant further investigation to understand their underlying regulatory mechanisms.

### 3.2. Target Gene Identification

Validated target genes for these differentially expressed miRNAs were then identified. The number of experimentally validated targets, identified using the multiMiR package, were the highest for SNU387 (582,432), followed by HepG2 (490,903), Caco-2 (295,398), and HCT116 (228,692) (shown in Table 1). The gene targets were further subjected to pathway analysis and gene ontology analysis.

### 3.3. Pathway Analysis

**Figure 2:**
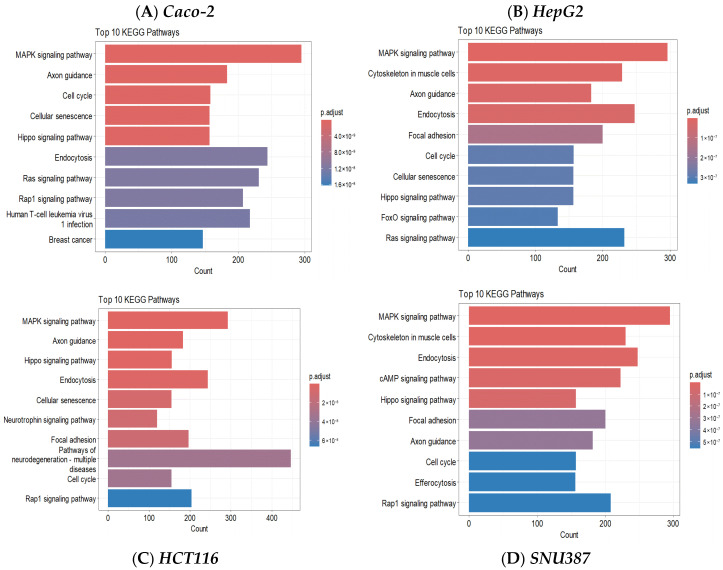
**KEGG pathway enrichment analysis for differentially expressed miRNAs.** Each panel shows top 10 significantly enriched pathways (adjusted *p* < 0.05) for each cell line. (**A**): **Caco-2**; (**B**): **HepG2**; (**C**): **HCT116**; (**D**): **SNU387**. The *x*-axis indicates the number of genes in each pathway, and color intensity represents adjusted *p*-values.

The gene targets obtained from target identification steps were further analyzed using the *enrichKEGG()* function from the *clusterProfiler package* to identify biological pathways enriched following TiO_2_ treatment. As shown in Figure 2, several KEGG pathways were commonly enriched across all four cell lines, namely MAPK signaling pathway, Axon guidance, Cell cycle, Hippo signaling pathway, and Endocytosis. Cellular senescence was overrepresented in Caco-2, HepG2 and HCT116 whereas Focal adhesion was overrepresented in HepG2, HCT116 and SNU387. Rap1 was overrepresented in Caco-2, HCT116, and SNU387. This shows that TiO_2_ exposure modulates key pathways involved in cell growth, proliferation, and differentiation, as well as transportation.

### 3.4. Gene Ontology Analysis

**Figure 3:**
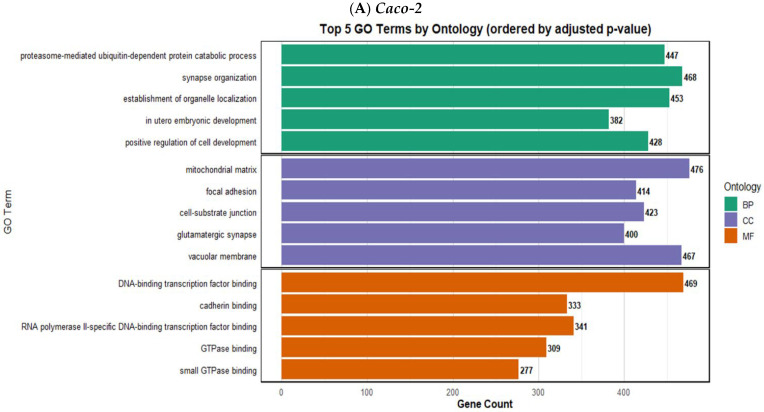

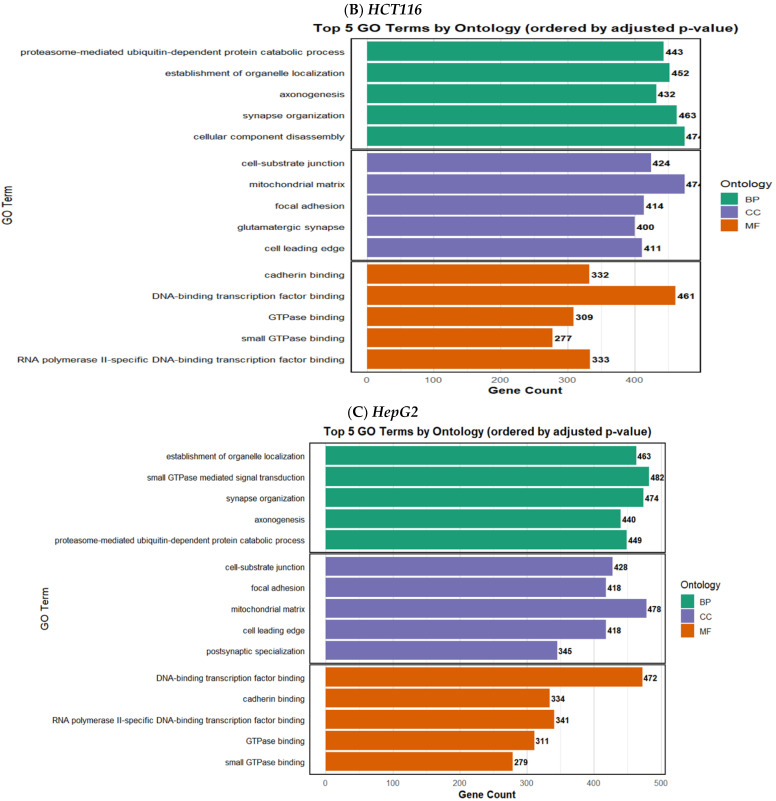

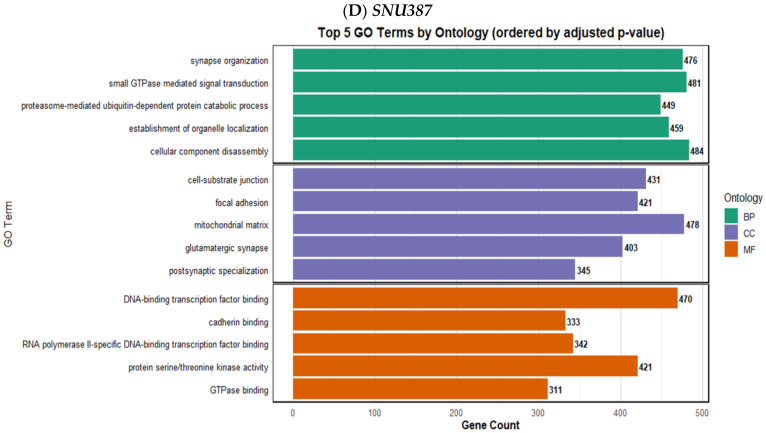
**Gene ontology (GO) enrichment analysis of validated target genes of differentially expressed miRNAs following TiO_2_ treatment in four human cell lines**. The bar plots display the top 5 enriched GO terms in each ontology category—Biological Process (BP), Cellular Component (CC), and Molecular Function (MF)—for each cell line. Bars are ordered by adjusted *p*-value, with gene count indicated at the end of each bar. (**A**) **Caco-2**, (**B**) **HCT116**, (**C**) **HepG2**, (**D**) **SNU387**. Distinct GO terms related to protein degradation, cell adhesion, transcription factor binding, and mitochondrial function were identified, suggesting both shared and cell-specific miRNA-mediated regulatory effects of TiO_2_ exposure.

Gene ontology enrichment analysis revealed both shared and cell line-specific enriched functions across the four cell lines after treatment with TiO_2_ (Figure 3). In the Biological Process (BP) category, the *proteasome-mediated ubiquitin-dependent protein catabolic process*, *synapse organization*, and *establishment of organelle localization* were common among all four cell lines, suggesting that modulation of protein turnover, neuronal development pathways, and structural organization is commonly affected by TiO_2_, regardless of cell line. Other commonalities included *axonogenesis* (HCT116 and HepG2), *cellular component disassembly* (HCT116 and SNU387), *and small GTPase-mediated signal transduction* (SNU387 and HepG2). Within Cellular Component (CC), *enrichment of the mitochondrial matrix, focal adhesions, and cell–substrate junctions* (all CC terms) across all four cell lines indicates impacts on cellular energy metabolism, adhesion, and synaptic structures. HCT116 and HepG2 shared a common *cell leading edge*; SNU387 and HepG2 shared common *postsynaptic specializations*. In the Molecular Function (MF) category, *RNA polymerase II-specific DNA-binding transcription factor binding*, *cadherin binding*, *GTPase binding*, and *DNA-binding transcription factor binding* were among the top-enriched terms, highlighting roles in transcriptional regulation, cell adhesion, and signal transduction. *Small GTPase binding* was a shared molecular function of HCT116, HepG2, and Caco2. These results demonstrate that miRNAs responsive to TiO_2_ affect genes vital to basic cellular processes, including protein degradation, cell organization, and signaling, with common and unique GO terms identified across colon and liver cell types.

## 4. Discussion

A major epigenetic mechanism is miRNA expression. Given the involvement of aberrant miRNA expression in the pathogenesis of an increasing number of disorders and diseases, interest is growing in assessing possible epigenetic toxicity induced by nanomaterials as an important approach in better understanding the potential risks for human health posed by these materials. Exposure to model TiO_2_ nanoparticles (including P25) has been shown to alter miRNA expression in several test systems, including HCT116 cells [35,36,37,38,39,40,41,42,43,44,45], and in one study, E171 (food-grade TiO_2_) was found to alter miRNA expression in human AGS human cells [46]. However, assessment of a TiO_2_ product, which humans could be exposed to in their diet, as well as in their use of personal care products, has not been well studied. This study examined the impact of exposures of this nanomaterial on miRNA expression in four cell lines relevant to human exposures.

In evaluating the effect of exposure to the TiO_2_ nanomaterial on miRNA expression in the cell lines, microarray analysis showed changes in miRNA expression in both the colorectal cells (Caco-2 and HCT116) and liver cells (HepG2 and SNU387). The number of differentially expressed miRNAs varied among the cell lines with a greater number of miRNAs upregulated than downregulated (as shown in Table 1). For several of these differentially expressed miRNAs, their altered expression has been associated with various colon or liver diseases [48,49,50]. These include miR-10b, miR-18a, miR-19a, miRNA-21, miRNA-29b, miRNA-34a, miRNA-93, miR-96, miRNA-100, miR-101, miRNA-106a, miR-135b, miR-203, miRNA-210, miR-221, miRNA-222, and miR-224. For example, miR-18a, which regulates many genes involved in cell cycle, proliferation, differentiation, response to different kinds of stress, apoptosis, and autophagy, has been observed to be overexpressed in many cancers, including colorectal and liver cancers [51,52].

In other examples of these miRNAs, similar results have been observed. miR-21 is involved in various biological processes, including T-cell activation and differentiation, and is often upregulated in cancer, influencing cell proliferation, apoptosis, and invasiveness [53]. Participating in various cellular processes, including cell proliferation, and angiogenesis, and apoptosis [54], miR-210 has been found to be up-regulated in almost all types of examined cancer types [55]. Abnormal expression of miR-221 has been shown to be involved in various tumor initiation and progression [56]. miR-221 is upregulated in hepatocellular carcinoma and promotes liver cancer cell proliferation and tumor angiogenesis. It is also elevated in colorectal cancer and has a role in tumor progression and metastasis. miRNA-224 plays a role in tumor development and progression by binding to multiple genes, which are involved in cell apoptosis, proliferation, invasion, migration, and autophagy. Studies have found that miRNA-224 had significantly higher expression in cancers, such as colon and hepatocellular carcinoma [57,58,59,60,61].

To explore the possible mechanisms of potential miRNA-dependent nanotoxicity, bioinformatics analysis was conducted. The functional enrichment analysis of target genes predicted for the differentially expressed miRNAs provides insights into the biological processes and pathways potentially regulated by these miRNAs. KEGG pathways enriched following TiO_2_ treatment were identified in the four cell lines. The Top 10 pathways in each cell line are shown in Figure 2. Interestingly, five of these pathways were common to all four cell lines. These KEGG pathways were also found enriched in RAW264.7 cells treated with model TiO_2_ nanoparticles (P25) [38]. Each of these pathways is known to be associated with diseases. The KEGG MAPK (mitogen-activated protein kinase) signaling pathway has a central role in many cellular physiological processes, including cell proliferation, differentiation, migration, stress response, and apoptosis; it involves a series of kinases that transmit signals from the cell surface to the nucleus [62,63]. This pathway has been well investigated in cancer studies, as abnormalities in MAPK signaling have a critical role in the development of cancer, including liver cancer [64,65]. The MAPK signaling pathway also plays a significant role in other liver diseases, particularly in the development and progression of conditions, such as alcoholic liver disease (ALD) and non-alcoholic fatty liver disease (NAFLD), and is involved in other aspects of liver disease, including hepatocyte apoptosis and fibrosis [66,67,68]. Additionally, the MAPK signaling pathway has a crucial role in the development and progression of colon cancer [69]. In a recent study, genes have been identified within the MAPK signaling pathway that were significantly dysregulated in colorectal cancer tissue when comparing carcinoma with adjacent normal tissue [70].

The KEGG axon guidance pathway is primarily associated with neuronal development in which developing axons find their way to their correct targets in the nervous system, ensuring the formation of accurate and functional neural circuits [71]. However, its dysregulation is emerging as a potential factor in liver disease progression; axon guidance cues can influence liver cell behavior and tissue development [72]. This pathway also has a role in colon cancer development and progression, particularly related to metastasis and tumor subtype identity. In one study, the KEGG axon guidance pathway was found to be significantly associated with poor overall and relapse-free survival, as well as with positive nodal status and metastasis in a cohort of patients with colon adenocarcinoma [73]. The KEGG Hippo pathway is key in tissue homeostasis, cell-fate decision, and control of organ size by regulating cell proliferation, differentiation, and apoptosis [74,75]. However, dysregulation of this pathway can lead to uncontrolled cell growth and has been associated with many malignancies [76,77]. Disruption of the Hippo signaling pathway leads to several diseases including inflammation and fibrosis [78]. In colon cancer and liver tumorigenesis, the Hippo pathway is also often dysregulated [79,80,81].

The KEGG endocytosis pathway is a crucial process for cell function whereby cell surface proteins, lipids, and fluid from the extracellular environment are packaged, sorted, and internalized into cells [82]. Abnormal endocytosis can lead to uncontrolled cell growth and proliferation, thereby contributing to cancer development, including liver and colon cancers [83]. The KEGG cell cycle pathway illustrates the progression of a cell through its various phases (G1, S, G2, and M), highlighting the role of cyclin-dependent kinases (CDKs) in driving the progression through the cell cycle and the checkpoints that ensure proper cell cycle progression, including the G1/S checkpoint, G2/M checkpoint, and the spindle assembly checkpoint [84]. Its dysregulation can lead to uncontrolled proliferation and potentially cancer development [85]. In several studies utilizing datasets to identify genes differentially expressed between tumor and normal tissue, bioinformatic analysis has revealed that the KEGG cell cycle pathway is significantly activated in hepatocellular carcinoma and in colorectal cancer [86,87,88].

Gene ontology enrichment analysis was also performed revealing the enriched functions (in the three categories) in the four cell lines after treatment with TiO_2_, as shown in Figure 3. Using GO and pathway enrichment methods, dysregulation of immune response, oxidative stress, MAPK signaling, cell cycle control, transport/endocytosis, membrane organization and several cancer-related pathways was reported in a study investigating the effects of E171 in Caco-2 cells at the transcriptome level [89]. This transcriptomic evidence aligns with our miRNA-based KEGG and GO analysis where we found MAPK signaling, cell cycle, endocytosis/transport, stress response, and disease-associated pathways being affected. Determining linkages between the GO functions and the specific KEGG pathways should be an important area of future investigation in providing valuable additional insight into ways in which the TiO_2_ nanomaterial may exert potential harmful effects.

Oxidative stress may be involved in the observed effects of exposure to the TiO_2_ product on miRNA expression. Although most studies implicating oxidative stress induced by ROS in the toxicity of TiO_2_ have been conducted with TiO_2_ nanoparticle models [90], reports have now shown that food-grade TiO_2_ can also induce oxidative stress [8,16,91,92]. Growing studies have demonstrated that ROS formation plays a significant role in regulating miRNA expression, impacting miRNA biogenesis, stability, and function. ROS can influence miRNA expression through various mechanisms, including the modification of transcription factors and epigenetic changes [93,94]. ROS can activate stress-related transcription factors, including p53, NF-κB, and HIF1α, which in turn can regulate the expression of miRNA genes. For example, ROS can induce the expression of miR-21 through the activation of NF-κB [95], or miR-210 through the activation of HIF1α [96]. Further studies will, however, be needed to better understand the causal pathways connecting nanomaterial exposure and altered miRNA expression.

The use of cancer-derived cell lines has limitations in that there can be biological and genetic differences with normal cells. However, using these cell lines is widely accepted in studying the effects of nanomaterials, favored for their low cost and reproducibility. In at least one recent study comparing nanomaterial toxicity testing in cancer-derived and normal cell lines, results suggested that several tests could be assessed using cancer-derived cell lines [97]. Additionally, future studies incorporating protein-level validation will be necessary to establish direct mechanistic causality between TiO_2_ exposure and activation. Our results are meant to offer potential involvement or predicted pathway activation, thereby providing guidance for these studies.

## 5. Conclusions

In conclusion, findings from this study demonstrate the impact of exposure to a TiO_2_ product on miRNA expression in human cells, supporting the potential involvement of this epigenetic mechanism in the possible biological effects of the TiO_2_ product. In each of the four cell lines, differentially expressed miRNAs were identified, including those predicted to play a role in regulating pathways associated with human diseases. Further studies will be needed to better define the intricate mechanisms of miRNA-mediated gene regulation and signaling involved, providing avenues for further exploration into their functional significance in biological processes and disease pathways. A better understanding of the epigenetic effects of TiO_2_ products should promote risk assessment and safe use practices of this type of material.

## Figures and Tables

**Table 1:** Summary of the number of differentially expressed miRNAs for each cell line and targets identified using multiMiR.

Cell Line	Number of miRNAs	Upregulated	Downregulated	Targets
Caco-2	94	91	3	295,398
HepG2	97	91	6	490,903
HCT116	53	43	10	228,692
SNU387	112	102	10	582,432

## Data Availability

The original contributions presented in this study are included in the article. Further inquiries can be directed to the corresponding author.

## References

[1] Vance M.E., Kuiken T., Vejerano E.P., McGinnis S.P., Hochella M.F., Rejeski D., Hull M.S. (2015). Nanotechnology in the real world: Redeveloping the nanomaterial consumer products inventory. Beilstein J. Nanotechnol..

[2] Musial J., Krakowiak R., Mlynarczyk D.T., Goslinski T., Stanisz B.J. (2020). Titanium dioxide nanoparticles in food and personal care products-what do we know about their safety?. Nanomaterials.

[3] US Food and Drug Administration (2005). Titanium Dioxide. Code of Federal Regulations, Title 21, Section 73.575.

[4] Weir A., Westerhoff P., Fabricius L., Hristovski K., von Goetz N. (2012). Titanium dioxide nanoparticles in food and personal care products. Environ. Sci. Technol..

[5] Peters R.J.B., van Bemmel G., Herrera-Rivera Z., Helsper H.P.F.G., Marvin H.J.P., Weigel S., Tromp P.C., Oomen A.G., Rietveld A.G., Bouwmeester H. (2014). Characterization of titanium dioxide nanoparticles in food products: Analytical methods to define nanoparticles. J. Agric. Food Chem..

[6] Bachler G., von Goetz N., Hungerbuhler K. (2015). Using physiologically based pharmacokinetic (PBPK) modeling for dietary risk assessment of titanium dioxide (TiO_2_) nanoparticles. Nanotoxicology.

[7] Dudefoi W., Moniz K., Allen-Vercoe E., Ropers M.-H., Walker V.K. (2017). Impact of food grade and nano-TiO_2_ particles on a human intestinal community. Food Chem. Toxicol..

[8] Hwang J.-S., Yu J., Kim H.-M., Oh J.-M., Choi S.-J. (2019). Food additive titanium dioxide and its fate in commercial foods. Nanomaterials.

[9] Putra C., Bello D., Tucker K.L., Kelleher S.L., Mangano K.M. (2022). Estimation of titanium dioxide intake by diet and stool assessment among US healthy adults. J. Nutr..

[10] Rompelberg C., Heringa M.B., van Donkersgoed G., Drijvers J., Roos A., Westenbrink S., Peters R., van Bemmel G., Brand W., Oomen A.G. (2016). Oral intake of added titanium dioxide and its nanofraction from food products, food supplements and toothpaste by the Dutch population. Nanotoxicology.

[11] Geraets L., Oomen A.G., Krystek P., Jacobsen N.R., Wallin H., Laurentie M., Verharen H.W., Brandon E.F., de Jong W.H. (2014). Tissue distribution and elimination after oral and intravenous administration of different titanium dioxide nanoparticles in rats. Part. Fibre Toxicol..

[12] He L., Wang H., Duan S., Gao Y., Lyu L., Ou X., Yu N., Zhang Y., Zheng L., Wang Y. (2022). Characterization of titanium dioxide nanoparticles in confectionary products and estimation of dietary exposure level among the Chinese population. NanoImpact.

[13] Bischoff N.S., Bussi M.R., Van Breda S.G., Jolani S., Sijm D.T.H.M., de Kok T.M., Briedé J.J. (2025). Food-grade titanium dioxide exposure between age groups and in global regions: A systematic review and meta-analysis. Crit. Rev. Food Sci. Nutr..

[14] Winkler H.C., Notter T., Meyer U., Naegeli H. (2018). Critical review of the safety assessment of titanium dioxide additives in food. J. Nanobiotechnol..

[15] Yang Y., Doudrick K., Bi X., Hristovski K., Herckes P., Westerhoff P., Kaegi R. (2014). Characterization of food-grade titanium dioxide: The presence of nanosized particles. Environ. Sci. Technol..

[16] Dorier M., Béal D., Marie-Desvergne C., Dubosson M., Barreau F., Houdeau E., Herlin-Boime N., Carriere M. (2017). Continuous in vitro exposure of intestinal epithelial cells to E171 food additive causes oxidative stress, inducing oxidation of DNA bases but no endoplasmic reticulum stress. Nanotoxicology.

[17] Baranowska-Wójcik E., Gustaw K., Szwajgier D., Oleszczuk P., Pawlikowska-Pawlęga B., Pawelec J., Kapral-Piotrowska J. (2021). Four types of TiO_2_ reduced the growth of selected lactic acid bacteria strains. Foods.

[18] Proquin H., Rodríguez-Ibarra C., Moonen C.G.J., Ortega I.M.U., Briedé J.J., de Kok T.M., van Loveren H., Chirino Y.I. (2017). Titanium dioxide food additive (E171) induces ROS formation and genotoxicity: Contribution of micro and nano-sized fractions. Mutagenesis.

[19] Dudefoi W., Terrisse H., Richard-Plouet M., Gautron E., Popa F., Humbert B., Ropers M.-H. (2017). Criteria to define a more relevant reference sample of titanium dioxide in the context of food: A multiscale approach. Food Addit. Contam. Part A.

[20] Ropers M.-H., Terrisse H., Mercier-Bonin M., Humbert B., Janus M. (2017). Titanium Dioxide as Food Additive. Applications of Titanium Dioxide.

[21] Bischoff N.S., Undas A.K., van Herwijnen M., Verheijen M., Briede J.J., van Breda S.G., Siim D.T.H.M., de Kok T.M. (2025). E171-induced toxicity in human iPSC-derived colon organoids: Effects on cell viability, ROS generation, DNA damage, and gene expression changes. Toxicol. In Vitro.

[22] Brand W., Peters R.J.B., Braakhuis H.M., Maślankiewicz L., Oomen A.G. (2020). Possible effects of titanium dioxide particles on human liver, intestinal tissue, spleen and kidney after oral exposure. Nanotoxicology.

[23] Bischoff N.S., de Kok T.M., Sijm D.T., van Breda S.G., Briedé J.J., Castenmiller J.J., Opperhuizen A., Chirino Y.I., Dirven H., Gott D. (2021). Possible Adverse Effects of Food Additive E171 (Titanium Dioxide) Related to Particle Specific Human Toxicity, Including the Immune System. Int. J. Mol. Sci..

[24] Baranowska-Wójcik E., Szwajgier D., Winiarska-Mieczan A. (2022). A review of research on the impact of E171/TiO_2_ NPs on the digestive tract. J. Trace Elem. Med. Biol..

[25] Felsenfeld G. (2014). A brief history of epigenetics. Cold Spring Harb. Perspect. Biol..

[26] Ambros V. (2004). The functions of animal microRNAs. Nature.

[27] Bartel D.P. (2009). MicroRNAs: Target recognition and regulatory functions. Cell.

[28] Montgomery R.L., van Rooij E. (2010). MicroRNA regulation as a therapeutic strategy for cardiovascular disease. Curr. Drug Targets.

[29] Olive V., Jiang I., He L. (2010). mir-17–92, a cluster of miRNAs in the midst of the cancer network. Int. J. Biochem. Cell Biol..

[30] Raitoharju E., Lyytikäinen L.-P., Levula M., Oksala N., Mennander A., Tarkka M., Klopp N., Illig T., Kähönen M., Karhunen P.J. (2011). miR-21, miR-210, miR-34a, and miR-146a/b are up-regulated in human atherosclerotic plaques in the Tampere Vascular Study. Atherosclerosis.

[31] Wang F., Ma Y., Wang H., Qin H. (2017). Reciprocal regulation between microRNAs and epigenetic machinery in colorectal cancer. Oncol. Lett..

[32] Lim J.P., Baeg G.H., Srinivasan D.K., Dheen S.T., Bay B.H. (2017). Potential adverse effects of engineered nanomaterials commonly used in food on the miRNome. Food Chem. Toxicol..

[33] Gedda M.R., Babele P.K., Zahra K., Madhukar P. (2019). Epigenetic Aspects of Engineered Nanomaterials: Is the Collateral Damage Inevitable?. Front. Bioeng. Biotechnol..

[34] Yu J., Loh X.J., Luo Y., Ge S., Fan X., Ruan J. (2020). Insights into the epigenetic effects of nanomaterials on cells. Biomater. Sci..

[35] Halappanavar S., Jackson P., Williams A., Jensen K.A., Hougaard K.S., Vogel U., Yauk C.L., Wallin H. (2011). Pulmonary response to surface-coated nanotitanium dioxide particles includes induction of acute phase response genes, inflammatory cascades, and changes in microRNAs: A toxicogenomic study. Environ. Mol. Mutagen..

[36] Thai S.-F., Wallace K.A., Jones C.P., Ren H., Prasad R.Y., Ward W.O., Kohan M.J., Blackman C.F. (2015). Signaling Pathways and MicroRNA Changes in Nano-TiO_2_ Treated Human Lung Epithelial (BEAS-2B) Cells. J. Nanosci. Nanotechnol..

[37] Alinovi R., Goldoni M., Pinelli S., Ravanetti F., Galetti M., Pelosi G., De Palma G., Apostoli P., Cacchioli A., Mutti A. (2017). Titanium dioxide aggregating nanoparticles induce autophagy and under-expression of microRNA 21 and 30a in A549 cell line: A comparative study with cobalt(II, III) oxide nanoparticles. Toxicol. In Vitro.

[38] Sui J., Fu Y., Zhang Y., Ma S., Yin L., Pu Y., Liang G. (2018). Molecular mechanism for miR-350 in regulating of titanium dioxide nanoparticles in macrophage RAW264.7 cells. Chemico-Biological Interactions.

[39] Hathaway Q.A., Durr A.J., Shepherd D.L., Pinti M.V., Brandebura A.N., Nichols C.E., Kunovac A., Goldsmith W.T., Friend S.A., Abukabda A.B. (2019). miRNA-378a as a key regulator of cardiovascular health following engineered nanomaterial inhalation exposure. Nanotoxicology.

[40] Ndika J., Seemab U., Poon W.-L., Fortino V., El-Nezami H., Karisola P., Alenius H. (2019). Silver, titanium dioxide, and zinc oxide nanoparticles trigger miRNA/isomiR expression changes in THP-1 cells that are proportional to their health hazard potential. Nanotoxicology.

[41] Xu S., Sui J., Fu Y., Wu W., Liu T., Yang S., Liang G. (2020). Titanium dioxide nanoparticles induced the apoptosis of RAW264.7 macrophages through miR-29b-3p/NFAT5 pathway. Environ. Sci. Pollut. Res..

[42] Li W., Jia M.X., Deng J., Wang J.H., Zuberi Z., Yang S., Ba J., Chen Z. (2020). MicroRNA Response and Toxicity of Potential Pathways in Human Colon Cancer Cells Exposed to Titanium Dioxide Nanoparticles. Cancers.

[43] Hu M., Palić D. (2020). Role of MicroRNAs in regulation of DNA damage in monocytes exposed to polystyrene and TiO_2_ nanoparticles. Toxicol. Rep..

[44] Ballesteros S., Vales G., Velázquez A., Pastor S., Alaraby M., Marcos R., Hernández A. (2021). MicroRNAs as a Suitable Biomarker to Detect the Effects of Long-Term Exposures to Nanomaterials. Studies on TiO2 NP and MWCNT. Nanomaterials.

[45] Soltysova A., Ludwig N., Diener C., Sramkova M., Kozics K., Jakic K., Balintova L., Bastus N.G., Moriones O.H., Liskova A. (2024). Gold and titania nanoparticles accumulated in the body induce late toxic effects and alterations in transcriptional and miRNA landscape. Environ. Sci. Nano.

[46] Han H., Yang M., Yoon C., Lee G., Kim D., Kim T., Kwak M., Heo M.B., Lee T.G., Kim S. (2021). Toxicity of orally administered food-grade titanium dioxide nanoparticles. J. Appl. Toxicol..

[47] Wells C., Pogribna M., Sharmah A., Paredes A., Word B., Patri A.K., Lyn-Cook B., Hammons G. (2024). Exposure to a Titanium Dioxide Product Alters DNA Methylation in Human Cells. Nanomaterials.

[48] Li Y., Kowdley K.V. (2012). MicroRNAs in common diseases. Genom. Proteom. Bioinform..

[49] Wang D., Liu J., Huo T., Tian Y., Zhao L. (2017). The role of microRNAs in colorectal liver metastasis: Important participants and potential clinical significances. Tumour Biol..

[50] Sharma P.C., Gupta A. (2020). MicroRNAs: Potential biomarkers for diagnosis and prognosis of different cancers. Transl. Cancer Res..

[51] Grassi A., Perilli L., Albertoni L., Tessarollo S., Mescoli C., Urso E.D.L., Fassan M., Rugge M., Zanovello P. (2018). A coordinate deregulation of microRNAs expressed in mucosa adjacent to tumor predicts relapse after resection in localized colon cancer. Mol. Cancer.

[52] Wang X., Lu J., Cao J., Ma B., Qi F. (2018). MicroRNA-18a promotors hepatocellular carcinoma proliferation, migration, and invasion by targeting Bxcl2L10. OncoTargets Ther..

[53] Rhim J., Baek W., Seo Y., Kim J.H. (2022). From molecular mechanisms to therapeutics: Understanding micor-RNA-21 in cancer. Cells.

[54] Chan Y.C., Banerjee J., Choi S.Y., Sen C.K. (2012). miR-210: The Master Hypoxamir. Microcirculation.

[55] Khalilian S., Bijanvand A., Abedinlou H., Ghafouri-Fard S. (2023). A review on the role of miR-210 in human disorders. Pathol.-Res. Pract..

[56] Abak A., Amini S., Sakhinia E., Abhari A. (2018). MicroRNA-221: Biogenesis, function, and signatures in human cancers. Eur. Rev. Med. Pharmacol. Sci..

[57] Ma D., Tao X., Gao F., Fan C., Wu D. (2012). miR-224 functions as an onco-miRNA in hepatocellular carcinoma cells by activating AKT signaling. Oncol. Lett..

[58] Zhang Y., Takahashi S., Tasaka A., Zhang Y., Takahashi S., Tasaka A., Yoshima T., Ochi H., Kazuaki C. (2013). Involvement of micro-RNA-224 in cell proliferation, migration; invasion, and anti-apoptosis in hepatocellular carcinoma. J. Gastroenterol. Hepatol..

[59] Zhang G.-J., Zhou H., Xiao H.-X., Li Y., Zhou T. (2013). Up-regulation of miR-224 promotes cancer cell proliferation and invasion and predicts relapse of colorectal cancer. Cancer Cell Int..

[60] Li Q., Ding C., Chen C., Zhang Z., Xiao H., Xie F., Lei L., Chen Y., Mao B., Jiang M. (2014). miR-224 promotion of cell migration and invasion by targeting Homeobox D 10 gene in human hepatocellular carcinoma. J. Gastroenterol. Hepatol..

[61] Lan S.H., Wu S.Y., Zuchini R., Lin X.Z., Su I.J., Tsai T.F., Lin Y.J., Wu C.T., Liu H.S. (2014). Autophagy suppresses tumorigenesis of hepatitis B virus-associated hepatocellular carcinoma through degradation of microRNA-224. Hepatology.

[62] McCubrey J.A., Steelman L.S., Chappell W.H., Abrams S.L., Wong E.W.T., Chang F., Lehmann B., Terrian D.M., Milella M., Tafuri A. (2007). Roles of the Raf/ MEK/ERK pathway in cell growth, malignant transformation and drug resistance. Biochim. Biophys. Acta.

[63] Darling N.J., Cook S.J. (2014). The role of MAPK signalling pathways in the response to endoplasmic reticulum stress. Biochim. Biophys. Acta BBA—Mol. Cell Res..

[64] Dhillon A.S., Hagan S., Rath O., Kolch W. (2007). MAP kinase signalling pathways in cancer. Oncogene.

[65] Mehdizadeh A., Somi M.H., Darabi M., Jabbarpour-Bonyadi M. (2016). Extracellular signal-regulated kinase 1 and 2 in cancer therapy: A focus on hepatocellular carcinoma. Mol. Biol. Rep..

[66] Widjaja A.A., Singh B.K., Adami E., Viswanathan S., Dong J., D’aGostino G.A., Ng B., Lim W.W., Tan J., Paleja B.S. (2019). Inhibiting Interleukin 11 Signaling Reduces Hepatocyte Death and Liver Fibrosis, Inflammation, and Steatosis in Mouse Models of Nonalcoholic Steatohepatitis. Gastroenterology.

[67] Jeng K.-S., Lu S.-J., Wang C.-H., Chang C.-F. (2020). Liver Fibrosis and Inflammation under the Control of ERK2. Int. J. Mol. Sci..

[68] Fetoh M.E.A.-E., Helal G.K., Saleh I.G., Ewees M., ElShafey M., Elnagar M.R., Akool E.-S. (2020). Cyclosporin a activates human hepatocellular carcinoma (HepG2 cells) proliferation: Implication of EGFR-mediated ERK1/2 signaling pathway. Naunyn-Schmiedeberg’s Arch. Pharmacol..

[69] Fang J.Y., Richardson B.C. (2005). The MAPK signalling pathways and colorectal cancer. Lancet Oncol..

[70] Slattery M.L., Mullany L.E., Sakoda L.C., Wolff R.K., Samowitz W.S., Herrick J.S. (2018). The MAPK-signaling pathway in colorectal cancer: Dysregulated genes and their association with microRNAs. Cancer Inform..

[71] Lin L., Lesnick T.G., Maraganore D.M., Isacson O. (2009). Axon guidance and synaptic maintenance: Preclinical markers for neurodegenerative disease and therapeutics. Trends Neurosci..

[72] Chicherova I., Hernandez C., Mann F., Zoulim F., Parent R. (2023). Axon guidance molecules in liver pathology: Journeys on a damaged passport. Liver Int..

[73] Rokavec M., Horst D., Hermeking H. (2017). Cellular model of colon cancer progression reveal signatures of mRNAs, miRNAs, IncRNAs, and epigenetic modifications associated with metastasis. Cancer Res..

[74] Barry E.R., Camargo F.D. (2013). The Hippo superhighway: Signaling crossroads converging on the Hippo/Yap pathway in stem cells and development. Curr. Opin. Cell Biol..

[75] Piccolo S., Dupont S., Cordenonsi M. (2014). The biology of YAP/TAZ: Hippo signaling and beyond. Physiol. Rev..

[76] Harvey K.F., Zhang X., Thomas D.M. (2013). The Hippo pathway and human cancer. Natl. Rev. Cancer.

[77] Moroishi T., Hansen C.G., Guan K.-L. (2015). The emerging roles of YAP and TAZ in cancer. Natl. Rev. Cancer.

[78] Panciera T., Azzolin L., Cordenonsi M., Piccolo S. (2017). Mechanobiology of YAP and TAZ in physiology and disease. Natl. Rev. Mol. Cell Biol..

[79] Lam-Himlin D.M., Daniels J.A., Gayyed M.F., Dong J., Maitra A., Pan D., Montgomery E.A., Anders R.A. (2006). The hippo pathway in human upper gastrointestinal dysplasia and carcinoma: A novel oncogenic pathway. Int. J. Gastrointest. Cancer.

[80] Zender L., Spector M.S., Xue W., Flemming P., Cordon-Cardo C., Silke J., Fan S.-T., Luk J.M., Wigler M., Hannon G.J. (2006). Identification and validation of oncogenes in liver cancer using an integrative oncogenomic approach. Cell.

[81] Zhou D., Zhang Y., Wu H., Barry E., Yin Y., Lawrence E., Dawson D., Willis J.E., Markowitz S.D., Camargo F.D. (2011). Mst1 and Mst2 protein kinases restrain intestinal stem cell proliferation and colonic tumorigenesis by inhibition of Yes-associated protein (Yap) overabundance. Proc. Natl. Acad. Sci. USA.

[82] Doherty G.J., McMahon H.T. (2009). Mechanisms of endocytosis. Annu. Rev. Biochem..

[83] Banushi B., Joseph S.R., Lum B., Lee J.J., Simpson F. (2023). Endocytosis in cancer and cancer therapy. Natl. Rev. Cancer.

[84] Kanehisa M., Goto S. (2000). KEGG: Kyoto encyclopedia of genes and genomes. Nucleic Acids Res..

[85] Evan G.I., Vosden K.H. (2001). Proliferation, cell cycle and apoptosis in cancer. Nature.

[86] Yan H., Li Z., Shen Q., Wang Q., Tian J., Jiang Q., Gao L. (2017). Aberrant expression of cell cycle and material metabolism related genes contributes to hepatocellular carcinoma occurrence. Pathol.-Res. Pract..

[87] Yu C., Chen F., Jiang J., Zhang H., Zhou M. (2019). Screening key genes and signaling pathways in colorectal cancer by integrated bioinformatics analysis. Mol. Med. Rep..

[88] Zhu H., Ji Y., Li W., Wu M. (2019). Identification of key pathways and genes in colorectal cancer to predict the prognosis based on mRNA interaction network. Oncol. Lett..

[89] Proquin H., Jonkhout M.C.M., Jetten M.J., van Loveren H., de Kok T.M., Briedé J.J. (2019). Transcriptome changes in undifferentiated Caco-2 cells exposed to food-grade titanium dioxide (E171): Contribution of the nano- and micro-sized particles. Sci. Rep..

[90] Song B., Zhou T., Yang W., Liu J., Shao L. (2016). Contribution of oxidative stress to TiO_2_ nanoparticle-induced toxicity. Environ. Toxicol. Pharmacol..

[91] Jayaram D.T., Payne C.K. (2020). Food-grade TiO_2_ particles generate intracellular superoxide and altered epigenetic modifiers in human lung cells. Chem. Res. Toxicol..

[92] Vignard J., Pettes-Duler A., Gaultier E., Cartier C., Weingarten L., Biesemeier A., Taubitz T., Pinton P., Bebeacua C., Devoille L. (2023). Food-grade titanium dioxide translocates across the buccal mucosa in pigs and induces genotoxicity in an in vitro model of human oral epithelium. Nanotoxicology.

[93] Lu C., Zhou D., Wang Q., Liu W., Yu F., Wu F., Chen C. (2020). Crosstalk of MicroRNAs and Oxidative Stress in the Pathogenesis of Cancer. Oxidative Med. Cell. Longev..

[94] Carbonel T., Gomes A.V. (2020). MicroRNAs in the regulation of cellular redox status and its implications in myocardial ischemia-reperfusion injury. Redox Biol..

[95] Meng F., Henson R., Wehbe–Janek H., Ghoshal K., Jacob S.T., Patel T. (2007). MicroRNA-21 regulates expression of the PTEN tumor suppressor gene in human hepatocellular cancer. Gastroenterology.

[96] Chen Z., Li Y., Zhang H., Huang P., Luthra R. (2010). Hypoxia-regulated microRNA-210 modulates mitochondrial function and decreases ISCU and COX10 expression. Oncogene.

[97] Kim I.Y., Kwak M., Kim J., Lee T.G., Heo M.B. (2022). Comparative study on nanotoxicity in human primary and cancer cell. Nanomaterials.

